# Model-Based Tumor Growth Dynamics and Therapy Response in a Mouse Model of *De Novo* Carcinogenesis

**DOI:** 10.1371/journal.pone.0143840

**Published:** 2015-12-09

**Authors:** Charalambos Loizides, Demetris Iacovides, Marios M. Hadjiandreou, Gizem Rizki, Achilleas Achilleos, Katerina Strati, Georgios D. Mitsis

**Affiliations:** 1 Department of Electrical & Electronic Engineering & KIOS Research Center for Intelligent Systems & Networks, University of Cyprus, Nicosia, Cyprus; 2 Department of Biological Sciences, University of Cyprus, Nicosia, Cyprus; 3 Department of Biology, Massachusetts Institute of Technology, Cambridge, MA, United States of America; 4 Department of Bioengineering, McGill University, Montreal QC, Canada; Istituto Superiore di Sanità, ITALY

## Abstract

Tumorigenesis is a complex, multistep process that depends on numerous alterations within the cell and contribution from the surrounding stroma. The ability to model macroscopic tumor evolution with high fidelity may contribute to better predictive tools for designing tumor therapy in the clinic. However, attempts to model tumor growth have mainly been developed and validated using data from xenograft mouse models, which fail to capture important aspects of tumorigenesis including tumor-initiating events and interactions with the immune system. In the present study, we investigate tumor growth and therapy dynamics in a mouse model of *de novo* carcinogenesis that closely recapitulates tumor initiation, progression and maintenance *in vivo*. We show that the rate of tumor growth and the effects of therapy are highly variable and mouse specific using a Gompertz model to describe tumor growth and a two-compartment pharmacokinetic/ pharmacodynamic model to describe the effects of therapy in mice treated with 5-FU. We show that inter-mouse growth variability is considerably larger than intra-mouse variability and that there is a correlation between tumor growth and drug kill rates. Our results show that *in vivo* tumor growth and regression in a double transgenic mouse model are highly variable both within and between subjects and that mathematical models can be used to capture the overall characteristics of this variability. In order for these models to become useful tools in the design of optimal therapy strategies and ultimately in clinical practice, a subject-specific modelling strategy is necessary, rather than approaches that are based on the average behavior of a given subject population which could provide erroneous results.

## Introduction

Tumor initiation, growth and maintenance are highly dynamic processes that depend on many biological parameters, such as the tissue of origin, initiating mutations, acquired genetic abnormalities, angiogenesis, the immune response and the heterogeneity of the cellular compartments within the tumor [1]. The complexity of tumor growth dynamics presents an obstacle in attempts to study and model tumor response to available therapeutics, and ultimately hinders our efforts towards personalized cancer therapy.

Mathematical models describing the biological phenomena underlying cancer growth can synthesize existing knowledge and provide a framework for understanding the complex mechanisms involved, test different assumptions and provide insights into questions that cannot be addressed by clinical/experimental studies alone. Moreover, these models can be used as a powerful tool in the quest for optimal therapy administration.

Numerous mathematical models of cancerous tissue growth at different levels, from gene expression to the phenomenological description of macroscopic tumor development, have been formulated. These include spatially and physiologically structured, continuous and agent-based, deterministic and stochastic, as well as phenomenological and mechanistic models [2–11]. Several studies have utilized ordinary differential equations (ODEs); in particular, the Gompertz growth model has been widely used [2] as it takes into account the reduced growth rate of the tumor that is observed as its size increases, unlike other models such as exponential growth models. Alternative ODE models have also been employed (e.g., proliferation quiescence models [3,4]).

Mathematical models have also been considered in the context of cancer treatment design, including chemotherapy [2,3,7], immunotherapy [5], as well as combination of both [6,12]. In most of these studies, the reported findings were not validated with clinical or experimental data, which is a critical step in assessing the usefulness of a proposed model in a clinical setting. On the other hand, validation has been performed in some studies using experimental data from xenograft mouse models [9,13–15].

However, there are several disadvantages to using xenograft mouse models to measure and predict tumor growth dynamics. Xenografts are transplanted in immunodefficient mice, which lack B and T lymphocytes and thus are incapable of initiating an immune response [16,17]. The immune system plays a pivotal role during cancer development, and it can negatively or positively regulate tumor growth, depending on the cancer subtype, tumor vascularization and other factors [18–22]. Thus, modeling tumor evolution using xenografts ignores an important variable, which affects tumor growth and contributes to the uncertainty of the predicted tumor state and size.

In addition, since tumors in immunodeficient mice are transplanted from already established tumor masses from “source” animals, the dynamics of the initial phase of tumor initiation and growth are not observed and consequently would not contribute to any attempt to quantitatively describe tumor growth. Moreover, cell lines used to establish these tumors carry a large number of genetic alterations, including chromosomal abnormalities such as deletions, duplications and inversions, i.e. characteristics that often appear rather late during tumor development. Refined versions of xenograft models which employ grafted human tumors as opposed to cell lines [23], are superior in many respects yet still miss these initial stages of tumor growth. Furthermore the limitations of obtaining source material for tumor xenografts make them difficult to use at a large scale.

In order to address some of these limitations, we use immunocompetent E6/E7 double transgenic mice [24,25] treated with DMBA/TPA [26] in the present study. In these mice, tumors arise in situ from normal skin as a result of chemical mutagenesis, and develop naturally in the context of a full immune system, thus more closely resembling the complexity and dynamics of tumorigenesis *in vivo*. Using data obtained from these mice, we use mathematical models to describe tumor progression as well as pharmacokinetic models to describe tumor response to 5-FU, a widely-used anti-cancer agent [27]. Specifically, we use a Gompertz model to describe tumor growth and a two-compartmental pharmacokinetic/ pharmacodynamic model to describe therapy effects, extracting biologically relevant parameters that characterize the observed behaviors and their variability. Our results demonstrate clearly and for the first time to this extent to our knowledge, that tumor growth and the effects of therapy in animal models of native cancer initiation and growth are highly variable and strongly subject-specific. They also suggest that this variability can be captured with subject-specific mathematical models. In turn, this implies that individualized, rather than nonspecific, modeling approaches yield promise for wide translation into clinical practice, with the ultimate goal of personalized optimal therapy design.

## Materials and Methods

**Fig 1:**
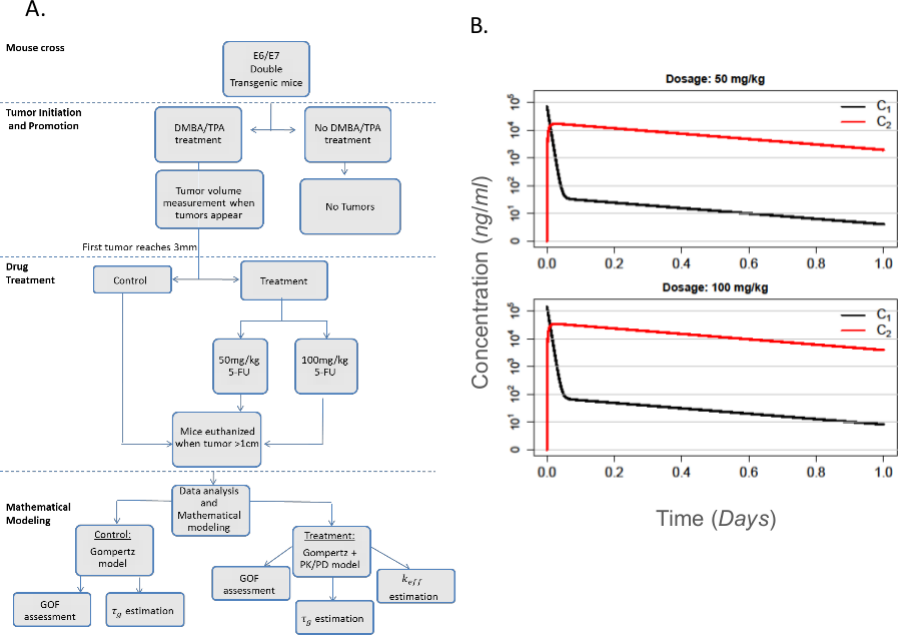
Experimental design and pharmacokinetic model: A. Schematic outline of the experiment. B. Plasma (*C*_*1*_) and tumor site (*C*_*2*_) drug concentrations for the two 5-FU dosages administered in the experiment. The figures show the concentrations from the time when the drug was administered until 24 hours later and are calculated according to Eq (5).

For this study, we used E6/E7 double transgenic mice that were either treated with DMBA/TPA or left untreated as a control (Fig 1A). Tumor volume measurements were initiated when the first tumor became visible, and treatment with 5-FU was initiated when the first tumor reached 3mm in diameter. A group of DMBA/TPA-treated animals did not receive 5-FU and were used as a control throughout the study. Tumor-bearing mice were euthanized when the largest tumor reached 1cm in diameter, and data collected was analyzed as described elsewhere.

### Ethics Statement

This study was carried out in strict accordance with the recommendations in the Guidelines for the Protection of Laboratory Animals of the Republic of Cyprus. The animal facility is licensed by the Veterinary Services (Republic of Cyprus Ministry of Agriculture and Natural Resources), the government body in charge of approving and overseeing laboratory animal work in Cyprus (license number CY.EXP.105) and the protocol was approved by the same authority (License number CY/EXP/PR.L1/2013). K14E6 and K14E7 mice are currently in use in the lab and are housed in the animal facility of the Department of Biological Sciences at the University of Cyprus. The mice were housed in individually ventilated cages (IVC), in pathogen free conditions. The work which we describe here does not involve procedures requiring anesthesia. Mice were sacrificed at the end-point unless health problems developed, which precluded the humane sustenance of the mouse to life. For example, in the case of tumor development, mice were sacrificed if the tumor reached a size larger than 1cm in diameter or if the tumor became ulcerated. We adhere to acceptable euthanasia guidelines using a CO2 chamber. Thus we ensure that our protocols are refined in order to minimize the discomfort of the animals.

### Breeding and Genotyping

K14E6hK14E7h mice were obtained by crossing K14E6H females with K14E7h heterozygous females. The mice are kept on a pure FVB genetic background. The presence of E6 and E7 transgenes was confirmed through PCR genotyping, using DNA extracted from mouse tail with Sigma DNA extraction Kit (Sigma-Aldrich, cat. # G1N10). E7 transgene was detected using (Oligo2/ E6TTL) primers, and E6 was detected using (K14709–4/E7TTL) primers as previously described [28]. PCR reactions were carried out using KAPATaq (Kappa Biosystems, cat. # KK1015).

### DMBA/TPA Treatment

DMBA/TPA treatment was initiated when mice became 7–8 weeks old. 200 μl of 0.03 μmol/μl DMBA (Sigma-Aldrich, cat. # D3254) was administered in a single dose on the back of mice previously shaved with electric clippers. After two weeks, mice were treated biweekly with 2.5 μg TPA (Sigma-Aldrich, cat. #P8139) diluted in 200 μl acetone. TPA treatment was continued twice a week for the whole duration of the experiment, until a mouse was sacrificed. Both chemicals were administered locally by skin painting.

### Tumor Measurements

Measurements of tumor volume on a mouse were initiated when the first tumor became visible, reaching 1–2 mm in diameter. We measured the volumes of 3 randomly selected tumors on each experimental animal. For each tumor and time point, we measured the length and width three times and subsequently used the median value of these three measurements to calculate the tumor volume, in order to reduce the effects of measurement error. Measurements were performed using a measuring caliper, twice a week. Tumor volume (*V*) was calculated using the formula:

$$V=\frac{\pi }{6}{\left(xy\right)}^{\frac{3}{2}}$$ (1)

where *x* and *y* denote the length and width of the tumor respectively. The formula assumes that the tumor has an ellipsoid shape [29]. Mouse weight was also recorded biweekly throughout the experiment. Note that we also used different shape assumptions to calculate tumor volume (rectangular, spheroid and an alternative ellipsoid approximation [29]) but the results were very similar to the patterns described below in all cases. Therefore, we show results obtained under the above widely used approximation (Eq (1)).

### Drug Treatments

When the first tumor on a mouse reached 3–4 mm in size, drug treatment with 5-Fluorouracil (5-FU; Sigma-Aldrich, cat. # F6627) was initiated. The drug was dissolved in 10% DMSO and administered weekly through intraperitoneal injection, in order to avoid the potential pitfalls of variable drug diffusion inherent to local application. Drug doses used were 50mg/kg (denoted 5-FU 1) or 100mg/kg (5-FU 2). Drug treatments were continued once a week, until a tumor reached ~1cm in diameter, at which point the mouse was sacrificed. The controls only received the drug delivery vehicle (DMSO).

### Mathematical Modeling

The main objective of our modeling approach is twofold. First, we investigate whether the Gompertz model, which has been widely used to describe tumor growth dynamics in experiments involving *in vitro* tumors and xenografts, can adequately describe the growth of *in vivo* tumors in mouse models of *de novo* carcinogenesis both without and during treatment. Second, we extract two biologically interpretable parameters (growth rate and drug kill rate) in order to quantitatively describe the growth dynamics of untreated tumors as well as the regression dynamics of tumors treated with 5-FU.

#### Tumor Growth Model

During the initial stages of tumorigenesis, cancer cells typically proliferate in an exponential fashion. As the tumor size increases, limitation in nutrient supply and/or mechanical constraints slows down its growth and the tumor eventually reaches a plateau size [30]. Unlike the simple exponential model [31], the Gompertz model (Eq (2)) is able to capture this behavior. The Gompertz model has been widely used due to its simplicity, its ability to describe experimental data better than alternative logistic models [32] as well as due to that it can incorporate the effect of therapy in a straightforward manner:

$$\frac{dT(t)}{dt}=\frac{1}{\tau_{g}}ln\left[\frac{ln(\theta_{g}/T_{0})}{ln(\theta_{g}/2T_{0})}\right]T(t)ln\left(\frac{\theta_{g}}{T(t)}\right)-L(T(t),C_{2}(t)),$$ (2)

where *T(t)* is the tumor volume (*mm* ^3^) at time *t*, *θ*_*g*_ is the plateau size (*mm* ^3^), *τ*_*g*_ is the tumor doubling time (*days*) and *T*_0_ is the initial tumor size. The first term in the right hand side (r.h.s.) represents the increase in cells due to proliferation while the second term in the r.h.s. is a function (*L*) used to describe the decrease in cells due to therapy, where the units are adjusted to yield macroscopic tumor volume under the approximation 10^6^cells ≈ 1*mm* ^3^ [30]. When *L* is 0, Eq (2) represents the untreated cancer model. It has been shown that anti-cancer drugs kill tumor cells by first-order kinetics [33]. Also, we assume a non-cycle specific drug action, i.e. the fraction of tumor cells killed by a drug of fixed concentration is not dependent upon the size of the tumor, in contrast to cycle-specific drugs, whereby the killed cell fraction depends on tumor growth fraction (and hence tumor size). Therefore, the function *L* is assumed to be linear in *T* [34]. The cell-loss term is an affine function of drug concentration at the tumor site, *C*_2_(*t*):

$$L(T(t),C_{2}(t))=k_{eff}(C_{2}(t)-C_{2,thr})H(C_{2}(t)-C_{2,thr})T(t),$$ (3)

where *C*_2,*thr*_ (*ng*/*ml*) is a therapeutic threshold under which the drug has no effect [35], *k*_*eff*_ (*ml*/(*days*•*ng*)) is the drug kill rate, and *H* is the Heaviside function:

H(C2(t)−C2,thr)={0,ifC2(t)<C2,thr1,ifC2(t)⩾C2,thr (4)

The Heaviside function *H* [36] is a discontinuous step function that takes the value 0 if the drug concentration *C*_2_(*t*) at any time *t* is below a threshold value *C*_2,*thr*_ (nullifying the effect of *L* in Eq (2)) and takes the value 1 if the concentration *C*_2_(*t*) is above *C*_2,*thr*_. Consequently, the drug will only be effective if its concentration reaches the threshold value. Below this value, it has no effect on tumor growth; however it would still contribute to overall drug toxicity.

#### Pharmacokinetic Model

The dynamic effects of drug administration can be described using pharmacokinetic (PK) models. The most straightforward method for modeling pharmacokinetics is to assume that the body can be approximated as a well-mixed tank. The advantage of this low-order model approach is the small number of parameters that can be estimated from experimental data. Low-order models are an adequate approximation for compounds with rapid distribution/ metabolism characteristics, the action of which is based on plasma concentration, or the treatment objective of which is pharmacokinetically-driven. On the other hand, in physiologically-based (PB) models [37] differential equations are used to describe concentrations in organs that are assumed well-mixed, and dynamics can be added as needed by subdividing organs into compartments. The number of parameters in a PB PK model is significantly higher than a compartmental PK description, and typically tissue-specific concentrations of the compound of interest are needed to estimate these parameters [38,39]. Due to this, PB PK models are used rather infrequently. In between these two extremes lie 2-compartmental models, which are more frequently used. A 2-compartmental model describing the kinetic behavior of the drug and its corresponding concentration profile is given by:

dC1(t)dt=k21C2(t)V2V1−k12C1(t)−k10C1(t)+d(t)V1dC2(t)dt=k12C1(t)V1V2−k21C2(t) (5)

where *C*_1_(*t*) and *C*_2_(*t*) represent the drug concentrations (in *ng*/*ml*) in the plasma and tumor site respectively, *V*_1_ and *V*_2_ represent the distributed volumes of the drug after the injection in the two compartments (in *ml*) and *d(t)* is the drug dosage (in *ng*/*day*). The rate constants *k*_12_ and *k*_21_ (in *days* ^-1^) correspond to the process that links the drug concentration in the plasma compartment, where the drug is introduced, and the drug concentration in the compartment of drug action at the cellular level. The rate constant *k*_10_ (also in *days* ^-1^) denotes other elimination processes. The parameters used in the PK model are taken from [13] (*k*_10_ = 151.2 *days* ^−1^, *k*_12_ = 5.62 *days* ^−1^, *k*_21_ = 2.31 *days* ^−1^, *V*_1_ = 0.71x10^3^ *ml*, *V*_2_ = 0.1x10^3^ *ml*). As these parameters are mostly drug-specific (rather than e.g. cancer-type specific), they are deemed adequate for use in our models.

In this study, two different doses of 5-FU were used. Using the equations described above, the kinetic behavior of 5-FU in the plasma (*C*_1_) and at the tumor site (*C*_2_) is determined for a 24 hour period following drug administration, as shown in Fig 1B.

#### Parameter Estimation

Intra- and inter-subject variability is a key characteristic of cancer. In this study, tumor growth dynamics, as well as response to treatment, were found to be variable from mouse to mouse, as well as within the same mouse. In order to obtain tumor-specific models, key parameters of the models were estimated using non-linear least squares estimation [40], implemented in the statistical programming language R (http://www.r-project.org). For the untreated mice, we estimated the doubling time of the tumor during exponential growth, *τ*_*g*_, whereas for the mice receiving treatment we estimated the tumor doubling time and the drug kill rate, *k*_*eff*_. In both cases, we employed the mathematical models described by Eqs (2), (3) and (5) above.

#### Goodness-of-Fit Assessment

The goodness-of-fit of the employed models was assessed using the Normalized Mean Square Error (NMSE), defined as follows:

Let *y*_1_,*y*_2_,…,*y*_*n*_ be the volume measurements for a tumor, at time points *t*_1_,*t*_2_,…,*t*_*n*_. Also let ${\hat{y}}_{1},{\hat{y}}_{2},\ldots ,{\hat{y}}_{n},$ be the tumor volume estimates predicted by the model at the same time points. The NMSE of the model output prediction is defined as:

$$NMSE=\frac{\sum_{i=1}^{n}{\left(y_{1}-{\hat{y}}_{i}\right)}^{2}}{\sum_{i=1}^{n}y_{i}^{2}}\times 100\%$$ (6)

Values of the NMSE that are close to 0 indicate a good fit of the model. The NMSE was chosen as a goodness-of-fit criterion since it is a standardized measure, independent of measurement units and scale.

## Results

### Model Goodness-of-Fit

Table 1 summarizes the NMSE values and their statistical properties for all tumors (treated and untreated case). These values were obtained from the untreated mice (8 mice, total 24 tumors) and the treated mice (16 mice, total 42 tumors). In the former case the NMSE between the measured data and the prediction achieved by the model of Eq (2) with *L*(*T*(*t*), *C*_2_(*t*)) = 0 was calculated, while in the latter case the NMSE between the measured data and the full model (Eq (2) with nonzero *L*, Eqs (3) and (5)) was calculated.

**Table 1:** Normalized mean squared error (NMSE) values for the fitted models with and without treatment. The values are percentages (%).

Treatment Group	Mean	Std. Err.	Min.	Q1	Median	Q3	Max.
Untreated (DMSO)	20.77	17.09	2.26	8.61	18.59	27.18	75.81
Treated (5FU)	26.25	20.08	2.47	13.31	19.11	35.33	88.17

Note that zero NMSE values are not expected due to the nature of the experimental data (macroscopic measurements) and the complexity of the underlying phenomena (see also Discussion and Conclusions). Overall, the NMSE values suggest that the Gompertz model yields a satisfactory fit to the tumor growth data. Note that values above 50% are observed in only one tumor for the untreated group (CM.77 T.2) and in five tumors for the treated group (CM.54 T.1 & T.2; CM.55 T.1; CM.62; T.1 & T.3).

### Tumor Growth Dynamics without Treatment

In order to estimate the parameters of the tumor growth model, we used data from a total of 24 tumors in 8 double transgenic mice (3 tumors each) in which carcinogenesis was induced with DMBA/TPA (as described in Materials and Methods, Eq (2)). In each case, the value of the tumor doubling time, *τ*_*g*_, was estimated from the respective tumor size time series. The numerical estimates are presented in Table 2.

**Table 2:** Numerical estimates of the tumor doubling time parameter, *τ*_*g*_, for the 8 mice in the DMSO (untreated) group. In each mouse three tumors were monitored. The doubling parameter is measured in days [*d*]. The values are more similar within mice (across columns) than between mice (across lines).

Mouse	Tumor 1	Tumor 2	Tumor 3
CM.37	6.650	8.256	9.868
CM.38	9.646	13.371	13.381
CM.53	37.922	24.312	23.510
CM.60	12.328	8.450	6.809
CM.76	27.251	55.120	19.439
CM.77	30.453	21.792	22.155
CM.78	13.865	9.475	11.645
CM.79	45.192	8.149	6.244

In our model, we set the parameter *θ*_*g*_ equal to 10^6^ *mm* ^3^, which corresponds to 10^12^ cells, considering that 1 *mm* ^3^ contains 10^6^ cells [30]. Considering the parameter *θ*_*g*_ unknown and estimating it from the experimental data led to unrealistically small maximum tumor capacities; therefore, we decided to keep a fixed value. Note that varying this value did not affect the results much, as long as it was considerably larger than the tumor size measurements, which is physiologically realistic. Furthermore, since the tumors in the collected data were found to be still in their early growth stage (roughly exponential growth), we also fitted an exponential model for comparison purposes, which yielded very similar findings to the Gompertzian model (see Discussion and Conclusions).

**Fig 2:**
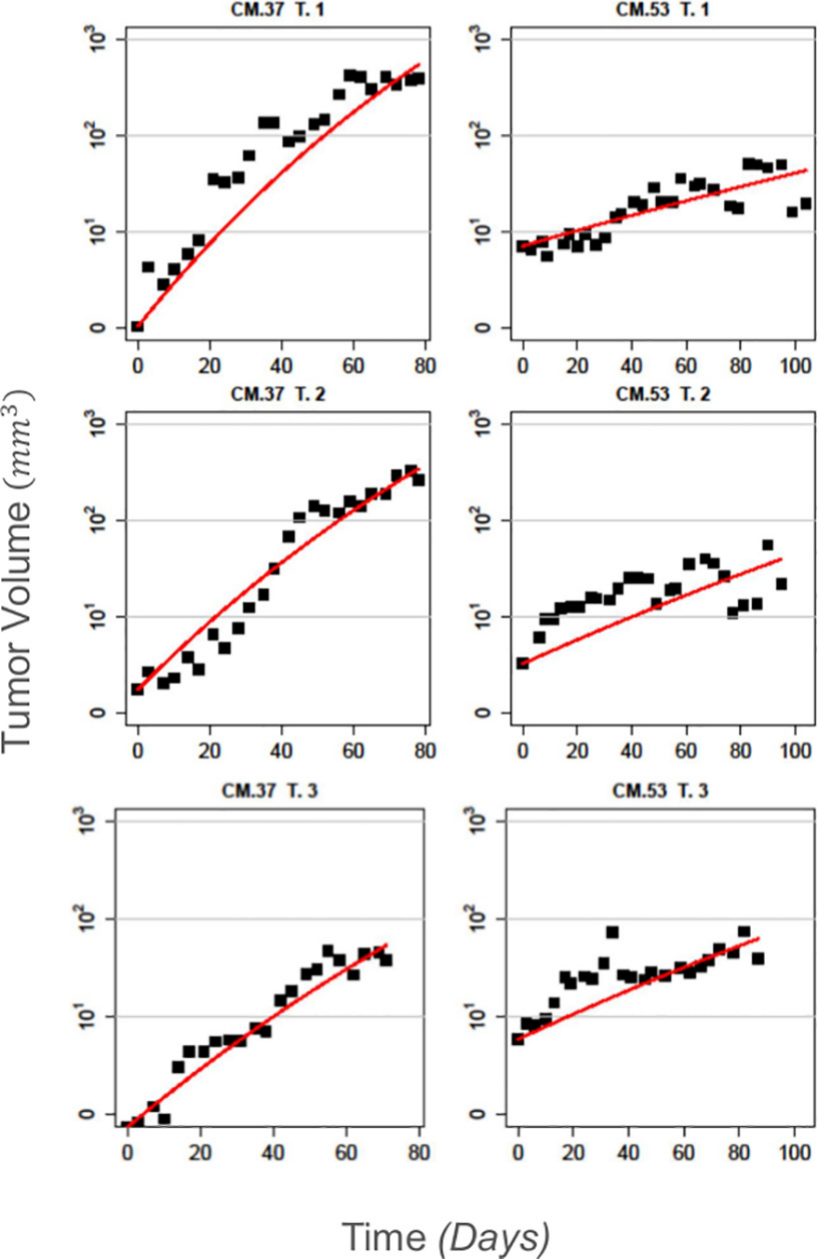
The variability of tumor growth *in vivo* can be captured with the Gompertz model: Indicative growth curves for a mouse with fast growing tumors (CM.37 –Left panel) and another with slow growing tumors (CM.53 –Right panel). The mice belong to the DMSO group and received no drug treatment. The black squares are the measured tumor volumes and the red line is the fitted model output using Eq (2) without treatment. The model provides an overall satisfactory fit to the data. The NMSE values are 17.4%, 5.3%, 10.7% for the three tumors of CM.37 and 16.1%, 27.1%, 19.8% for the three tumors of CM.53. Additionally tumor growth rate appears to be mouse specific.

The overall fit of the Gompertzian model was quantified using the NMSE between the model prediction and the tumor growth experimental data (Table 1). Fig 2 illustrates all three monitored tumors from two representative mice (CM.37 and CM.53), along with the fitted model curves (the curves from all tumors are given in Fig P2 in S1 Text). We can observe that the fitted curves follow the data closely, suggesting a satisfactory fit, which is further confirmed by the corresponding NMSE values (17.4%, 5.3%, 10.7% for CM.37 and 16.1%, 27.1%, 19.8% for CM.53). The NMSE achieved by the model, averaged over all tumors, was found to be 20.77±17.09% (mean ± standard error, Table 1).

It is evident that there exists a high degree of inter-and intra-mouse variability with respect to tumor growth. In some cases, tumors may exhibit rapid dynamics and grow to high volumes quickly (e.g. CM.37 T.1, *τ*_*g*_ = 6.65; almost 1000 *mm* ^3^ in ~80 days; on the other extreme lie tumors that exhibit slow growth (e.g. CM.53 T.3, *τ*_*g*_ = 23.51; less than 100 *mm* ^3^ in ~90 days). However, the results also imply that tumor growth dynamics are mouse-specific to an extent, as the intra-mouse variability of the parameter *τ*_*g*_ is considerably smaller than the corresponding inter-mouse variability, with tumors in the same mouse exhibiting more similar growth dynamics. This can be further observed in Fig 2 where we compare tumor growth in two mice, one with fast and another with slow growing tumors. In order to better understand the sources of the observed variability, we decomposed the total variation into its two components, i.e. within- and between- mouse variation. These sources can be estimated from the respective within and between mean square errors (MSW and MSB respectively). In our data, MSW = 118.0 and MSB = 282.9. The *F*-test that compares these two variance sources gives a *p*-value = 0.07, which is marginally significant.

**Fig 3:**
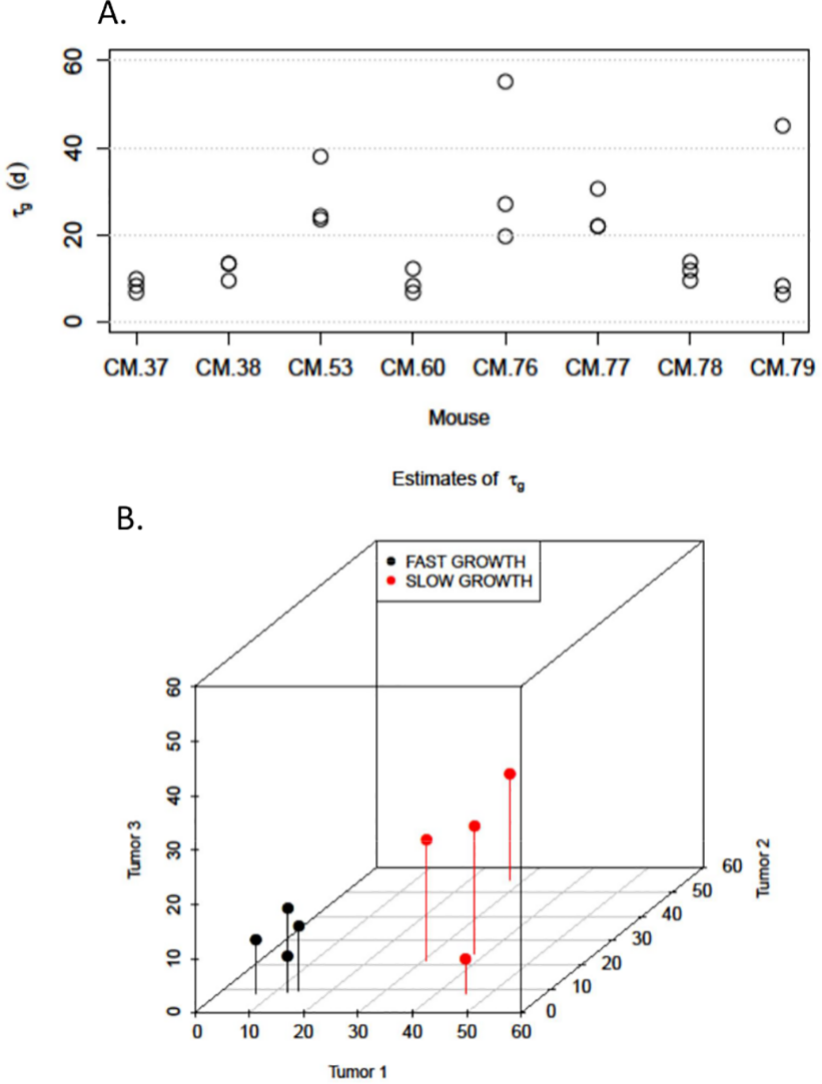
Tumor growth *in vivo* is mouse-specific: A. Top panel–Estimates of the tumor doubling time parameter *τ*_*g*_. For each of the eight mice in the DMSO group we obtain one estimate for each of the three monitored tumors. The figure suggests that tumor growth is mouse specific. Four mice (CM.37, 38, 60 & 78) exhibit three fast growing tumors while three mice (CM.53, 76 & 77) exhibit three slow growing tumors. The only exception is CM.79, for which the first tumor is slow growing, unlike the other two tumors. B. Bottom panel–The existence of two discrete growth rate groups is further illustrated by the distribution of all three tumor doubling times for the eight mice, shown here in a three-dimensional scatter plot. Mice CM.37, 38, 60 & 78 comprise one group, while the other four comprise a second group.

Fig 3A shows how the growth rate parameters *τ*_*g*_ are distributed among the 8 mice of the DMSO group. We can see that in four mice (CM.37, 38, 60 & 78) all three tumors grow at a relatively fast rate. On the other hand, in three mice (CM.53, 76 & 77) all three tumors exhibited a slow growth rate. These two groups appear to be distinct. Only one mouse (CM.79) exhibited a more variable growth pattern: one slow-growing tumor (*τ*_*g*_ = 45.19 days) and two fast-growing tumors (*τ*_*g*_ = 8.15 and *τ*_*g*_ = 8.24 days respectively). Therefore, despite the intra-mouse variability, it appears that tumor growth is mouse specific.

To further illustrate this, we plot all three *τ*_*g*_ estimates for each mouse in scatter form (Fig 3B). We observe that two distinct groups are formed. The first group is located very close to the origin of the axes and comprises four mice (CM.37, CM38, CM60 and CM.78), which exhibit fast growing tumors. The second group is located further from the origin and comprises the four remaining mice (CM.53, CM.76, CM.77, and CM.79), whose tumors have slower growth rates overall.

### Tumor Growth Dynamics with Treatment

**Fig 4:**
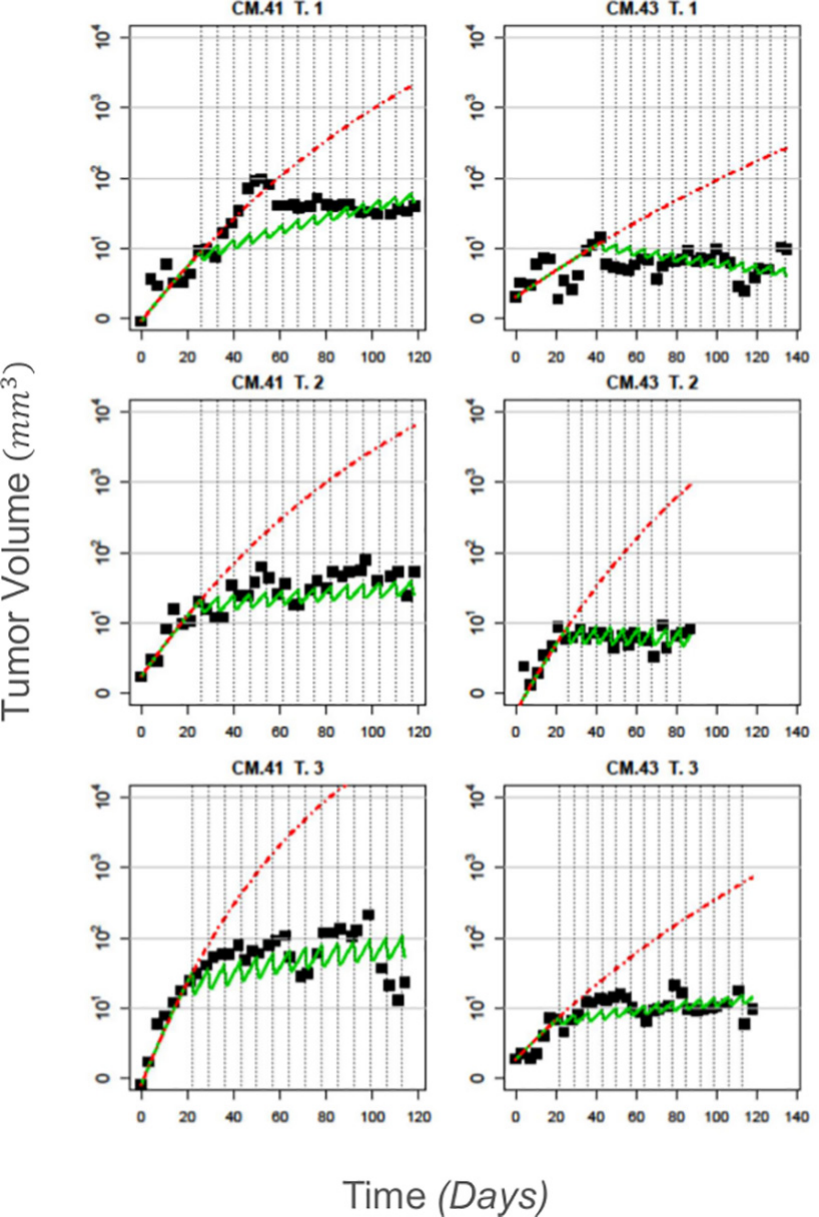
The response to 5-FU treatment can be captured with the combined Gompertz/two-compartment pharmacokinetic model: Indicative figures from two treated mice, one from each treatment group. Left panel–CM.41 from 5-FU 1; Right panel–CM.43 from 5-FU 2. The black squares indicate the measured tumor volumes while the green line is the fitted model based on Eqs (2–5). The red dashed line is the prediction of what would have happened if the tumor was left untreated based on the pre-treatment data estimates of the tumor growth rate. Our model appears to accurately capture, both the growth and treatment dynamics of the tumor. The NMSE values for the three tumors of CM.41 are 44.7%, 26.8% and 35.4% and for the three tumors of CM.43 these are 18.5%, 6.7% and 13.0% respectively.

In order to describe the observed behavior of the tumors after treatment with 5-FU, we applied the tumor growth and PK/PD models (Eqs (2), (3) and (5)). For all mice in the two 5-FU treatment groups, we used the data collected before the commencement of treatment in order to estimate *τ*_*g*_, as described above for the DMSO mice. The numerical estimates are given in Table 3. Representative examples of the model fit are shown in Fig 4 (the fitted curves from all treated tumors are presented in Figs P3 and P4 in S1 Text). The overall fit when we incorporated treatment in the model was found to be slightly worse than the fit achieved for the untreated mice. This is probably due to the inclusion of the PK model, for which the parameters were obtained from the literature [7], since their estimation requires specialized experiments. Therefore, despite the fact that the model has more parameters, the overall fit did not improve. However, the fit is still considered satisfactory, with an average NMSE equal to 26.2 ± 20.0% (Table 1). It should also be noted that for some of the tumors, there were not sufficient data available for *τ*_*g*_ to be estimated. These tumors were excluded from subsequent analysis since the treatment model assumes knowledge of the value of *τ*_*g*_ in order to estimate the drug kill rate parameter, *k*_*eff*_ (Table 3). Due to this, a total of 42 tumors from the 16 mice in the treated group were monitored. Furthermore, the estimated doubling time parameters for the tumors in the two 5-FU groups are in general smaller compared to the ones estimated from the DMSO group. Additional analysis showed that there is a positive correlation between the estimated value of *τ*_*g*_ and the number of observations used to obtain that estimate, which is also partly due to the fact that tumors grow faster during their initial growth phase (Section 3 in S1 Text). In turn, this implies that in the initial stages of their growth, tumors grow more uniformly and mouse-specific growth behavior emerges more clearly at later stages.

**Table 3:** Numerical estimates of the tumor doubling time *τ*_*g*_ and the drug kill rate *k*_*eff*_ for the mice tumors in the two treatment groups. For some tumors the estimation of *τ*_*g*_ and subsequently *k*_*eff*_ was not possible due to the lack of sufficient pre-treatment data. Further analysis showed that *k*_*eff*_ is less subject-dependent and more tumor-specific compared to *τ*_*g*_.

Mouse	Tumor	*τ*_*g*_ [d]	*k*_*eff*_ [*ml*/d ng]	Treatment Group
CM.41	1	7.373	5.02e-05	5-FU 1 (50mg/kg)
CM.41	2	6.574	7.52e-05	5-FU 1 (50mg/kg)
CM.41	3	3.704	1.16e-04	5-FU 1 (50mg/kg)
CM.42	1	13.531	5.51e-05	5-FU 1 (50mg/kg)
CM.42	2	5.012	7.19e-05	5-FU 1 (50mg/kg)
CM.42	3	4.664	5.37e-05	5-FU 1 (50mg/kg)
CM.43	1	15.726	2.29e-05	5-FU 2 (100mg/kg)
CM.43	2	5.911	4.59e-05	5-FU 2 (100mg/kg)
CM.43	3	10.613	2.32e-05	5-FU 2 (100mg/kg)
CM.49	1	16.540	2.07e-05	5-FU 2 (100mg/kg)
CM.49	2	60.396	2.40e-05	5-FU 2 (100mg/kg)
CM.49	3	18.766	1.05e-05	5-FU 2 (100mg/kg)
CM.54	1	22.832	1.00e-15	5-FU 1 (50mg/kg)
CM.54	2	11.206	3.95e-04	5-FU 1 (50mg/kg)
CM.54	3	13.679	2.29e-06	5-FU 1 (50mg/kg)
CM.55	1	9.639	3.36e-05	5-FU 1 (50mg/kg)
CM.55	2	5.683	7.87e-05	5-FU 1 (50mg/kg)
CM.55	3	4.349	9.12e-05	5-FU 1 (50mg/kg)
CM.56	1	11.252	1.25e-05	5-FU 2 (100mg/kg)
CM.57	1	23.716	2.54e-06	5-FU 2 (100mg/kg)
CM.57	2	9.045	2.33e-05	5-FU 2 (100mg/kg)
CM.57	3	5.192	4.99e-05	5-FU 2 (100mg/kg)
CM.62	1	8.920	2.43e-20	5-FU 1 (50mg/kg)
CM.62	2	12.109	1.72e-20	5-FU 1 (50mg/kg)
CM.62	3	87.771	3.62e-22	5-FU 1 (50mg/kg)
CM.63	2	8.993	2.30e-05	5-FU 2 (100mg/kg)
CM.66	1	3.136	1.27e-04	5-FU 1 (50mg/kg)
CM.66	2	5.844	7.04e-05	5-FU 1 (50mg/kg)
CM.67	1	7.851	2.69e-05	5-FU 2 (100mg/kg)
CM.67	2	12.429	1.36e-05	5-FU 2 (100mg/kg)
CM.73	1	11.134	8.24e-06	5-FU 2 (100mg/kg)
CM.73	2	3.672	4.75e-05	5-FU 2 (100mg/kg)
CM.73	3	4.734	4.83e-05	5-FU 2 (100mg/kg)
CM.84	1	20.589	1.47e-05	5-FU 2 (100mg/kg)
CM.84	2	13.684	1.23e-05	5-FU 2 (100mg/kg)
CM.84	3	13.352	6.43e-06	5-FU 2 (100mg/kg)
CM.90	1	6.288	9.47e-05	5-FU 1 (50mg/kg)
CM.91	1	4.792	5.89e-05	5-FU 2 (100mg/kg)
CM.91	2	13.441	1.07e-05	5-FU 2 (100mg/kg)

**Fig 5:**
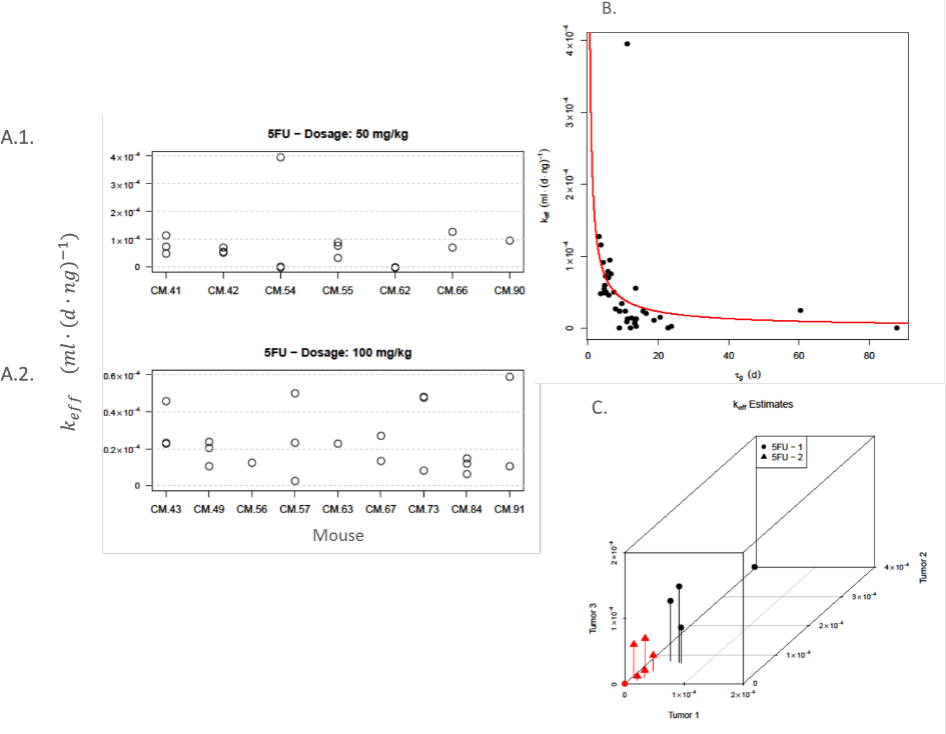
Drug efficiency is variable and depends on tumor growth rate. A. Left Panel–Estimates of the drug kill rate parameter, *k*_*eff*_, for the two treatment groups in our experiment (A.1.: 5-FU 1; A.2.: 5-FU 2). Unlike the tumor growth rate, which is appears mouse specific, response to treatment that is reflected through *k*_*eff*_ is less dependent on mouse. B. Top-right Panel: Scatterplot of *τ*_*g*_ against *k*_*eff*_ and non-parametric regression fit from kernel regression (red line). A negative correlation between the two parameters or equivalently, a positive correlation between growth and drug kill rates is observed, further supported by the value of Spearman’s correlation coefficient (*r* = -0.726). C. Bottom-right Panel: Scatter plot of the estimated values of the drug kill rate parameter (*k*_*eff*_) from all three tumors per mouse, for both treatment groups, Two distinct groups are formed, whereby the first group (black–more effective per unit drug volume) comprises four out of the five 5FU-1 mice and the second group (red–less effective per unit drug volume) comprises the remaining 5FU-1 mouse (CM.62) and all five 5-FU2 mice.

The parameter *k*_*eff*_ can be seen as a measure of drug efficacy per concentration unit. Unlike the tumor doubling time *τ*_*g*_, *k*_*eff*_ does not appear to be mouse specific (Fig 5A). There are a few examples of mice for which all tumors exhibit no response to treatment (CM. 62 for 5-FU 1, CM. 84 from 5-FU 2); however, in general there is much greater within mouse variation compared to *τ*_*g*_ (MSW = 4.74×10^−9^, MSB = 5.52×10^−9^ and *p*-value = 0.574). Several tumors from both treatment groups demonstrate minimal response to treatment (e.g. CM.54 T.3, group 5-FU 1 and CM.57 T.1, group 5-FU 2) while others exhibit a significant decrease in their growth rates (e.g. CM.41 T.3, group 5-FU 1 and CM.91 T.1, group 5-FU 2). We also observe a strongly nonlinear negative correlation between *τ*_*g*_ and *k*_*eff*_ (Fig 5B). Spearman’s *r* correlation coefficient, which is a measure of nonlinear correlation, was found to be *r* = -0.727, while the corresponding significance test yields a *p*-value = 6.42×10^−7^ which confirms the existence of the aforementioned correlation. Similar results were obtained after removing three possible outliers from our analysis (Fig 5B, *r* = 0.775, *p*-value = 3.98×10^−7^). This indicates that, for identical treatment regiments, tumors with faster growth rates exhibit a more pronounced response to treatment when compared to tumors with slow growth rates.

The variability in the values of *k*_*eff*_ appears to be mostly associated with the two treatment groups. The mice treated with the lower dose of 5-FU (5-FU 1) have on average larger values than the ones treated with the higher drug dose (5-FU 2). The *p*-value for the two-sided *t*-test is *p* = 0.035, which provides strong evidence that the difference in the parameter value between the two groups is statistically significant. This is further illustrated in Fig 5C, where we show the distribution of the drug kill rate parameter *k*_*eff*_ for the mice for which estimates from all three tumors were available. There are five such mice from each group. The results reveal that the mice may be divided in two groups, according to the effectiveness of the drug per volume unit. The lower dosage is more effective per drug volume unit and the first (more effective) group is formed exclusively from mice belonging in the 5-FU 1 group (four out of five mice). The second (less effective) cluster is formed by the remaining mouse from 5-FU 1 and the five mice from the 5-FU 2 treatment group. This suggests that even though a higher drug dose may have a greater net impact on the overall tumor size, the lower dose is essentially more effective per unit of drug used.

## Discussion and Conclusions

Mathematical models can become valuable tools in our future arsenal of cancer treatment by allowing us to better predict tumor growth and response to treatment. However, most modeling tools are developed in the context of serious limitations. Since lack of access to longitudinal data of human tumor growth is unlikely to be overcome anytime soon for most cancer types, modeling of tumors from experimental animals, most predominantly mice, is the best alternative. Most modeling up to now has been performed with data from xenograft models utilizing cancer cells or cell lines injected subcutaneously in mice. These models have allowed the initial development and evaluation of modeling strategies. However, the limitations of xenografts in recapitulating the constraints of natural tumor growth, hinder the potential of clinical tools developed using such data.

In order to address these shortcomings, we have utilized data from a mouse model of skin carcinogenesis using transgenic animals that express the HPV16 oncogenes E6 and E7 in the target tissue [25]. Use of the skin as a model lends itself to relatively easy monitoring of tumor growth while preserving the advantages of *de novo* tumor generation. Such models develop tumors with a slower rate of kinetics. In order to expedite the carcinogenic process and facilitate the collection of a larger volume of longitudinal data, we locally applied to the skin the tumor initiator DMBA and promoter TPA. An added advantage of using this carcinogenesis system is that it has been extensively studied and characterized in the past [26]. While this tumor model is not representative of all types of tumor growth, it is an important tool towards improving our current understanding of *in vivo* tumor growth dynamics, and mathematical modeling using this system represents novel work to our knowledge. In contrast to the usually employed xenograft models, animals are immunocompetent, which yields tumor growth subject to constraints such as a functional immune system and tissue restrictions. Moreover, since tumor formation is initiated *de novo*, our experimental model incorporates tumor growth dynamics during the early stages of tumorigenesis, which is not the case when xenografts are used.

The results presented in this study suggest that the employed models are able to capture the variability in the growth dynamics of untreated tumors, as well as their post-treatment evolution. They also reveal that: (i) tumor growth is highly variable in *de novo* tumor animal models as compared to xenograft models [9] (ii) For drug-free tumor growth, the inter-mouse variability is considerably larger than the intra-mouse variability, yielding two distinct groups of mice that exhibit slow and fast growing tumors (iii) Drug efficiency is also variable, and it is more tumor-dependent than subject-specific. Collectively, these results highlight the variability of cancer progression and emphasize the importance of pursuing subject and/or tumor-specific modeling approaches in order to quantify tumor growth dynamics. Only such approaches yield promise for eventually being incorporated in clinical practice in the context of optimal therapy design, subject to further refinements such as experimental monitoring of drug toxicity, modeling of drug resistance etc. [8]. Despite the variability in the growth and therapy response of the tumors, the employed models can successfully capture the underlying dynamics offering hope that even human tumors, which have the added complexity of heterogeneous genetic backgrounds, may be amenable to modeling.

Our results provide strong evidence that tumor growth rate is subject specific (Table 2 and Fig 3A). Almost all tumors recorded for each individual mouse exhibit similar growth rates and there appears to be a distinction between mice with fast and mice with slow growing tumors. Tumors from the former group exhibited tumor doubling time around or less than 10 days while tumors from the latter group exhibited a doubling time of more than 20 days. The mice used were bred on a pure genetic background chosen to minimize preexisting genetic variation among subjects at the time of tumor initiation. However, there remain differences between individual mice which may lead to the type of subject specificity observed. Genetic rearrangements and intrinsic variation during immune system maturation may contribute to a source of genetic variation among different mice [16]. Alternatively, or in addition, genetic variation due to stochastic (vs. inherited) mutations may account for the observed differences. Also note that these results were replicated even when we repeated our analysis using an exponential growth model, since most of the experimental data appear to be in the early stages of approximately exponential growth. The exponential model resulted in relatively faster growth rate estimates (Gompertz: average *τ*_*g*_ = 18.5 days, SE = 2.65 days; Exponential: average *τ*_*g*_ = 11.6 days, SE = 0.87 days); however, the distinction of the mice in two groups (fast and slow growth rates) remained, with the same mice in each group. Therefore, we present results obtained using the more physiologically realistic Gompertz model.

Furthermore, subject specificity may be linked to epigenetic differences or other non-genetic differences between subjects leading to phenotypic heterogeneity. For example, this could include sporadic mutations developed in a mouse during stages of skin development. Interestingly, mouse-specific differences were found to arise more clearly during the later stages of tumor growth, whereas the observed behavior was more uniform during earlier growth stages (Section 3 in S1 Text). These increased differences seen during the later stages of tumor growth may be reflective of the increased accuracy of size quantification at these stages (due to larger overall tumor volumes). Alternatively, they may be attributed to the fact that the mouse- specific differences have a higher cumulative effect since they precede tumor-specific mutations and account for the observed pattern of variability.

Like many other chemotherapeutic agents, 5-FU is a drug expected to target faster growing tumors more efficiently due to its mechanism of action [41]. As anticipated, faster tumor growth was associated with a more pronounced response to treatment. This implies that growth kinetics analyzed with similar modeling efforts could be used to predict response to 5-FU or other similar agents in the future. Given the degree of similarity in growth rates within subjects it is difficult to make any conclusions regarding subject specificity of treatment.

All currently available mathematical models of cancer progression are bound by important limitations that are due to the fact that cancer is a very complex biological phenomenon that occurs on many levels, from the intracellular to the tissue/organism level [1,42]. As of yet, it is not feasible to obtain experimental data with the required time resolution on all these levels in order to fully validate dynamic cancer models, particularly multi-scale models, which are naturally the most principled approach for capturing these multi-level interactions. Therefore, any attempt to model cancer progression has to rely on incomplete information. The consequence of this is that cancer models fall in two categories. The first category includes simplified models that do not attempt to explicitly model the biological mechanisms that underlie cancer but are simple enough so that their parameters can be identified by data that are typically available [8,9,43]. The second includes spatiotemporal models that attempt to capture some of the underlying biological mechanisms but, due to the lack of experimental data, cannot be identified by the latter (i.e. they are not identifiable) and hence have to rely on prior assumptions about most model parameters. These include cellular automata and center models, whereby the former consider cells as lattice points occupying fixed grid points and assign probabilities of transition from one cell state to another, while the latter relax the fixed-point assumption and solve continuous time partial differential equations for each cell [10,11,44]. Here, we employ a model of the first type, as the only available measurements are that of macroscopic tumor size, as is the case in the vast majority of similar studies.

In this context, selecting the model structure that is well-suited to a particular application is of great importance. For example, in cases where information about the geometrical structure of the tissue is available, and if diffusion phenomena (of cells, locally produced molecules, or externally delivered drugs) are at stake, then spatially structured models could/should be used [45]. This is true for models of tumor invasion, in particular gliomas (brain tumors), that are known to evolve with radial diffusion from a localized point in brain, invading the surrounding tissue within the skull. However, often no clear space structure emerges and drug concentration may be taken in first approximation as spatially homogeneous in a given cell compartment. In some cases normal, tumor and stromal cells are not clearly compartmentalized; therefore, space is not always a relevant structure variable for model design in cancer growth. In these cases non-spatial models are a more suitable choice. Overall, there exists no universal cancer model that could be applied to all cases and model selection should be based on cancer type, the type of experimental data that may be available and the aim of each study.

Therefore, in the present study we selected a modeling approach that was suitable to the available experimental data and to our main aim, which was to investigate and quantify the variability in tumor growth and response to therapy that was observed in the studied *de novo* animal cancer model, and illustrate the importance of using subject and/or tumor-specific models. As our results suggest, growth rates are highly variable and relying on one growth rate estimate to predict another tumor’s behavior may lead to erroneous results. Models of the type employed herein may be combined in a straightforward manner with optimal therapy design strategies in order to design subject-specific protocols by e.g. estimating the tumor growth parameter *τ*_*g*_ for a given subject using pre-treatment data and using this estimate to predict growth during the administration of therapy [8,46]. Furthermore, more sophisticated stochastic modeling strategies may be used to predict tumor growth in an adaptive manner, starting from an average, prior model and continuously updating model parameters as measurements from a new subject are made available [9]. Alternatively, the relatively small observed within-subject variability of tumor growth parameters (compared to the respective between-subjects variability) can be utilized in more complex multidimensional models, whereby instead of considering individual tumors, the entire tumor load of the organism could be considered. In such models, for example, the tumor load could be modeled as a vector of individual tumors, with correlated growth parameters. Thus, any information obtained for one tumor can be utilized for the prediction of the others.

Furthermore, future models could be refined by modeling the dynamics of specific cancer cell subpopulations proposed to be important in growth, therapeutic response and disease relapse. It is now well established that cancer stem cells (CSCs) play a significant role in tumor development and maintenance. Therefore, they are perhaps the most important cellular compartment within a heterogeneous tumor cell population and should be considered in mathematical models designed to determine or predict tumor growth dynamics. Additionally, this is a population which may be refractory to a lot of conventional cancer treatments, especially those targeting rapidly proliferating cells. Stem cells evading rounds of therapy may re-initiate growth. Thus finding ways to model their specific dynamics may help in the development of better relapse-free therapy regimens. The ultimate goal would be to combine such models with optimal therapy design methods. Therefore, we are currently implementing a new set of experiments combining modeling with optimal and adaptive control, the potential advantages of which have been recently suggested [46], with an aim to design subject-specific treatments and validate their superiority over standard (e.g. metronomic) therapies.

## Supporting Information

S1 Text Supplementary material. (DOCX)
