# Supplementary material for: Model-Based Tumor Growth Dynamics and Therapy Response in a Mouse Model of De Novo Carcinogenesis
Source: PLoS One. 2015 Dec 9;10(12):e0143840. doi: 10.1371/journal.pone.0143840 (PMC4674149; doi:10.1371/journal.pone.0143840)
Supplement: S1 Text — (DOCX) [file pone.0143840.s001.docx]

Supplementary Material For “Model-based Tumor Growth Dynamics and Therapy Response in a Mouse Model of de Novo Carcinogenesis”

1. **Indicative pictures of the tumors in our experiments.**

**Fig P1:** Tumor growth and response to therapy is variable within a mouse, and among different animals. **A.** Tumor measurements were initiated when the first tumor became visible reaching 1-2 mm in diameter (i). When the first tumor reached 3-4mm in diameter, drug treatment was initiated (ii) until a tumor reached about 10mm in diameter (iii) at which point the mouse was sacrificed. **B.** H&E staining shows typical tumor pathology of DMBA/TPA- induced mouse papillomas.

**2. Tumor growth Gompertz model fit in untreated and treated mice**

In the main paper we present some indicative examples of the fitted growth curves on both the treated (DMSO) and untreated (5-FU 1 & 2) tumors. Here we present these curves for all tumors. Fig P2 shows the 24 tumors (8 mice x 3 tumors each) from the DMSO group. It is evident that growth rate strongly depends on the mouse, with each subject’s tumors exhibiting relatively similar growth behavior compared to tumors from different subjects. This is supported by more formal results in the main paper.


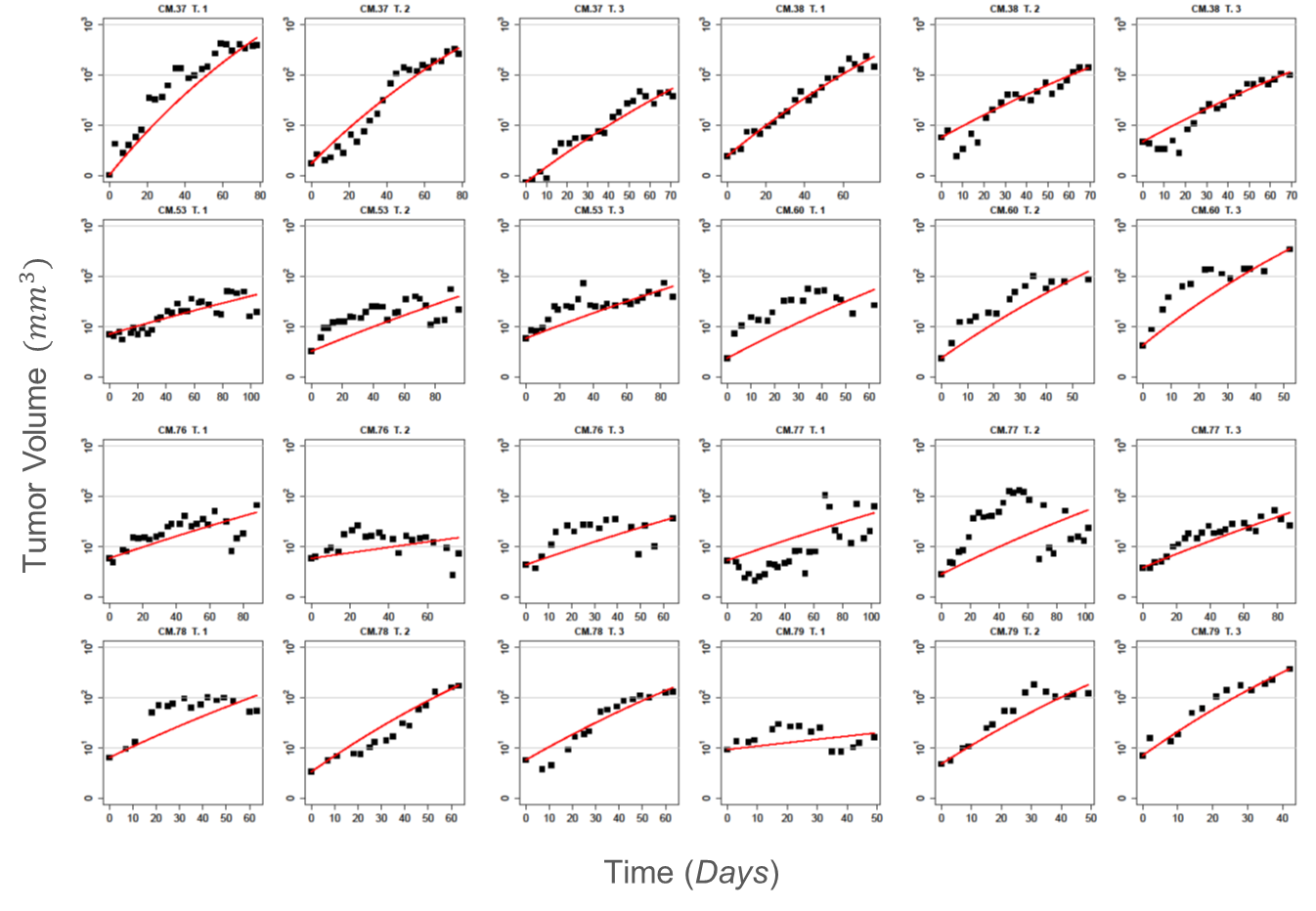


**Fig P2:** Experimental data (black squares) and fitted growth curves (red lines) from the tumors in the DMSO data. The estimates of the tumor doubling time for each tumor are presented in Table 1 of the main paper.

Figs P3 and P4 present the fitted curves for the low and high dosage 5-FU treatment groups respectively. Not all tumors in our experiment are presented, since for some a sufficient amount of pre-treatment data needed for fitting the model was not available.


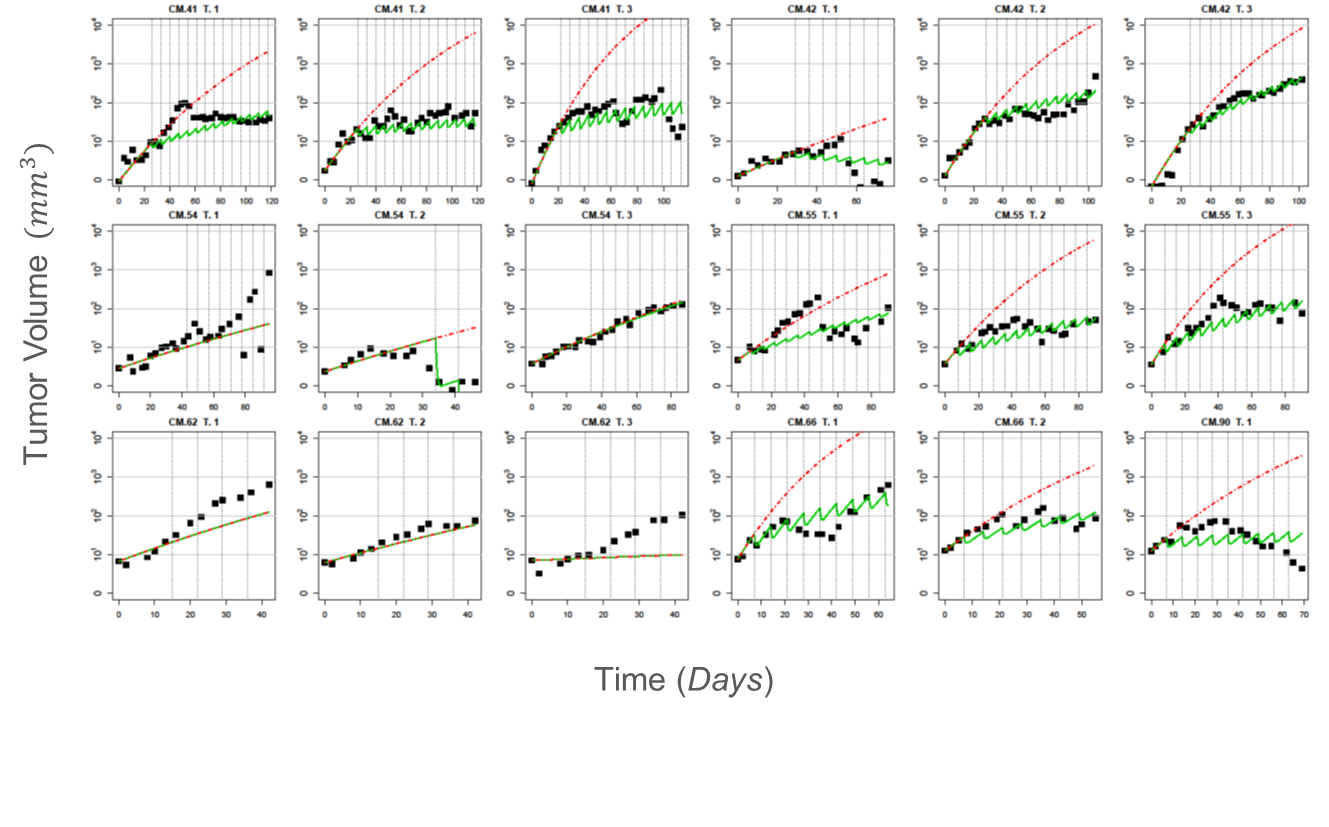


**Fig P3:** Experimental data (black squares) and fitted growth curves with treatment (green lines) for the 5-FU 1 data (low dosage). The dotted red lines show the estimated growth curve, had the tumors been left untreated. The vertical dotted lines show the treatment days. The numerical estimates of both the tumor doubling time and drug kill rate are presented in Table 2 of the main paper.


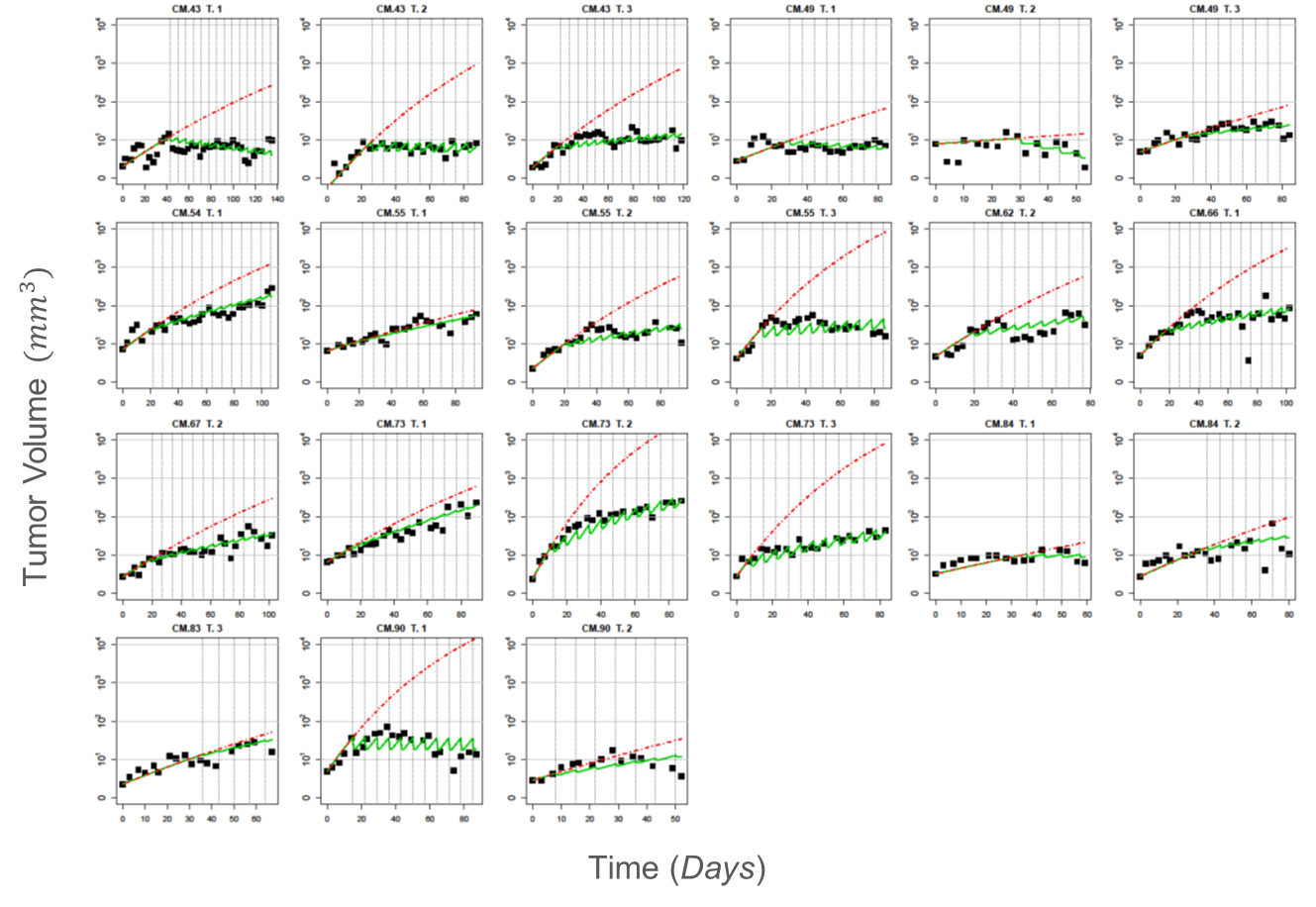


**Fig P4:** Experimental data (black squares) and fitted growth curves with treatment (green lines) for the 5-FU 2 data (high dosage). The dotted red lines show the estimated growth curve, had the tumors been left untreated. The vertical dotted lines show the treatment dates. The numerical estimates of both the tumor doubling time and drug kill rate are presented in Table 2 of the main paper.

**3. Tumor doubling time (**$\boldsymbol{\tau}_{\boldsymbol{g}}$**) estimates for the treated tumors (5FU 1 & 2).**

In order to apply the mathematical model that describes the effect of therapy (Eq. (2) in the main paper) on the data from the treated tumors, we first estimate their tumor growth parameters, $\tau_{g}$. To this end, we use the available data points before the commencement of treatment. Fig P5 shows these estimates for the two 5-FU groups. For some of the tumors in the two treatment groups, there were not enough data available before treatment for $\tau_{g}$ to be estimated, which is why some tumors and/or subjects are not presented in the figures. The estimates in this case appear to be less mouse specific compared to the respective estimates based on the DMSO data. Also, their values are on average smaller than the ones estimated from the DMSO data (i.e. faster growth). This is mostly due to the fact that in the early stages of their growth, tumors exhibit faster growth. Related to this, we identified a significant negative correlation between the value of $\tau_{g}$ estimates and the number of points used for estimation. Furthermore, this suggests that the emergence of mouse-specific tumor growth behavior becomes more evident at later growth stages, possibly due to the accumulation of tumor and/or mice specific mutations, whereas in the initial stages of their growth, tumors grow more uniformly. This was confirmed by repeating the analysis of the untreated mice using fewer data points, whereby the emerging growth patterns were found to be more uniform compared to using all data points (Fig 3A, main paper).


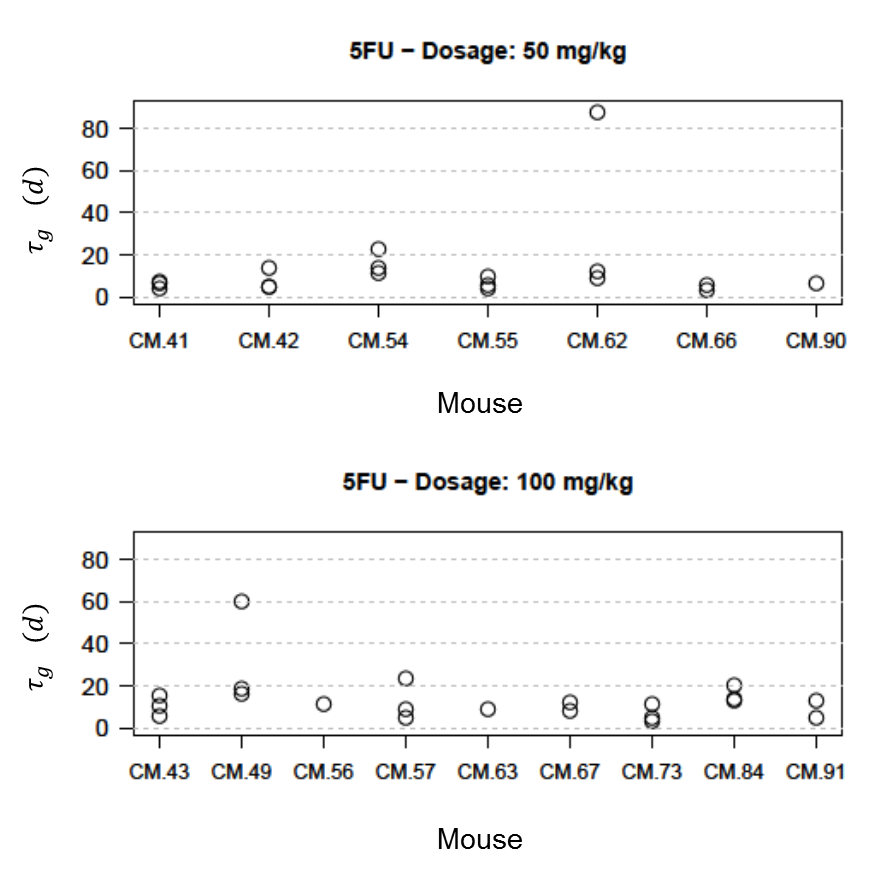


**Fig P5:** Estimates of the tumor doubling time ($\tau_{g}$) for the two treatment groups: 5-FU 1 (low dosage) – top; 5-FU 2 (high dosage) – bottom.
